# Selected health characteristics are associated with urban Canadians’ acceptability of policies promoting healthier restaurant food environments

**DOI:** 10.1017/S136898002400257X

**Published:** 2024-12-27

**Authors:** Jessica Lambert-De Francesch, Kadia Saint-Onge, Nazeem Muhajarine, Lise Gauvin

**Affiliations:** 1 Centre de Recherche du Centre Hospitalier de l’Université de Montréal, Montréal H2X 0A9, Québec, Canada; 2 École de santé publique, Département de Médecine Sociale et Préventive, Université de Montréal, Montréal H3N 1X9, Québec, Canada; 3 Département de kinésiologie, Faculté de médecine, Université de Laval, Québec, Canada; 4 Department of Community Health and Epidemiology, College of Medicine, University of Saskatchewan, Saskatoon, Saskatchewan, Canada; 5 Saskatchewan Population Health and Evaluation Research Unit, Saskatoon, Saskatchewan, Canada

**Keywords:** Public opinion, Policy, Restaurant, Health, Multilevel analysis

## Abstract

**Objective:**

The adoption of policies promoting healthier restaurant food environments is contingent on their acceptability. Limited evidence exists regarding individual characteristics associated with restaurant food environment policy acceptability, especially health-related characteristics. This study examined associations between health characteristics and restaurant food environment policy acceptability among urban Canadians.

**Design:**

Links between health characteristics and complete agreement levels with selected policies were examined using data in the cross-sectional *Targeting Healthy Eating and Physical Activity* survey study, that is, a large pan-Canadian study on policy acceptability. For each policy, several logistic multilevel regression analyses were conducted.

**Setting:**

Canada’s seventeen most populated census metropolitan areas.

**Participants:**

Urban Canadian adults responded to the survey (*n* 27 162).

**Results:**

Body mass index was not associated with acceptability after adjustments for other health and sociodemographic characteristics were made. Across all policies and analyses, those reporting excellent or very good health statuses were more likely to be in complete agreement with targeted policies than those with good health statuses. For selected policies and analyses, those reporting poor health statuses were also more likely to be in complete agreement than those describing their health status as good. For all policies and analyses, both those consuming restaurant-prepared foods daily and those never consuming these foods were more likely to be in complete agreement than those consuming these foods once per week.

**Conclusions:**

More research is needed to explain discrepancies in acceptability according to health characteristics. Bringing this study’s findings to the attention of policymakers may help build momentum for policy enactment.

By 2035, it is expected that the global prevalence of overweight and obesity will attain a new record: over 50 % of the population will have a body mass index (BMI) in the overweight (BMI ≥ 25 kg/m^2^) or obese (BMI ≥ 30 kg/m^2^) category^(1)^. A potent contributor to the rising overweight/obesity prevalence is the current urban food environment^(2)^, characterised by the omnipresence of fast-food and full-service restaurants. Common to both of these restaurant settings is the retailing of often calorie dense foods with low nutritional appeal^(3,4)^. Canadians are avid consumers of these highly palatable restaurant-prepared foods. Indeed, when asked how long it had been since Canadian study participants consumed their last restaurant-prepared food, 21·8 % of participants responded the previous day^(5)^. Similarly, Seale *et al.*’s (2022) study found that 48 % of Canadian participants had eaten food from a fast-food restaurant in the previous week^(6)^. In an era where consuming restaurant-prepared foods has become the new normal, rethinking certain aspects of the restaurant food environment (RFE) may be a critical step to halt rising overweight/obesity rates^(7,8)^. Implementing food policy interventions aimed at improving the healthfulness of the food environment, particularly the RFE, may help achieve these greater public health nutrition goals^(9–11)^.

Despite the plethora of studies highlighting the potential benefits of implementing selected RFE policy interventions, bringing these policies to the political agenda may be a challenging endeavour. One of these challenges relates to their public acceptability levels (i.e. agreement levels), a key consideration in the policy adoption process^(12)^. According to Kingdon’s *Multiple Streams Framework*, three streams (i.e. conditions) are required for a policy to be placed on the policy agenda, with the last condition directly related to policy acceptability^(13)^. These streams relate to the (1) recognition of a health-related problem by the public (e.g. high overweight/obesity prevalence), (2) identification of a solution to the problem (e.g. evidence-based RFE policy) and (3) presence of a favourable political climate (e.g. high public acceptability levels for the proposed RFE policy). When these three streams converge, a policy window is opened. The adoption of a RFE policy is then contingent on the work of policy entrepreneurs, described as ‘energetic actors who engage in collaborative efforts in and around government to promote policy innovations’^(14)^. These actors, who have high acceptability levels for the targeted RFE policy, will seize the opportunity related to the crossing of the three streams and bolster the policy agenda setting process thanks to their distinctive policy-influencing strategies.

Despite the importance of high policy acceptability levels during the policy agenda setting process, acceptability research has often been overshadowed by other implementation considerations^(15)^ and has only relatively recently attracted academic attention^(16)^. The latter statements are particularly salient when examining the sparse body of literature pertaining to the acceptability of RFE policies. An exception to this observation is a study by Lambert-De Francesch *et al.* (2024) who examined the sociodemographic characteristics associated with complete agreement levels for RFE policies among urban Canadians living in seventeen different census metropolitan areas (CMAs)^(17)^. In addition to determining gender-, age-, education-, income- and ethnicity-related differences in acceptability, these authors observed statistically significant differences in acceptability levels across the different CMAs. According to the same study, sociodemographic differences did not help explain CMA-level variance in acceptability levels, suggesting that other factors are responsible for explaining jurisdictional differences in RFE policy acceptability.

The broader food policy acceptability literature may help elucidate the nature of other factors underpinning RFE policy acceptability levels. Among these factors are individual health-related characteristics. Regarding the latter, it has, for example, been observed that individuals tend to express higher levels of acceptability for policies with lower personal impact^(15,18,19)^. In this sense, it would be expected that those who adhere to more healthful dietary practices would exhibit greater acceptability levels of policies promoting healthy eating since these measures would not interfere with their current dietary practices. The validity of this hypothesis has, however, not been substantiated by all^(20,21)^ and has rarely been tested specifically for RFE policies^(21)^. Other health-related correlates of food policy acceptability have also been studied, namely, BMI and, to a lesser extent, perceived health status. Regarding the former variable, at the current time, it is impossible to draw conclusions as to the direction and magnitude of associations between BMI and food policy acceptability, as positive, negative and even null associations have been observed^(15,21–24)^. In regard to the latter variable, which has been shown to be a reliable and valid predictor of overall health status^(25)^, given that less than a handful of studies have included this variable in their study, it is unwise to draw any conclusions on the nature of these associations^(21)^. In all, a more comprehensive examination is needed to elucidate if (and how) specific health-related variables are associated with RFE policy acceptability. This information would help better understand the favourability of the political climate towards RFE policies among population subgroups, a stepping stone in propelling the RFE policy agenda.

This study examines the associations between individual health characteristics and complete agreement levels of three RFE policies among urban-dwelling Canadians. The targeted RFE policies pertain to (1) offering healthier default side dish options on restaurant menus, (2) implementing fast-food zoning restrictions near schools and (3) eliminating unhealthy foods sold in municipal food outlets.

## Methods

### Study design, sampling and weighting

This study used the cross-sectional data available from the *Targeting Healthy Eating and Physical Activity* (THEPA) survey. The THEPA survey provided novel information on urban Canadians’ acceptability levels for forty-five built environment and policy interventions. These interventions concerned modifying the Covid-19 sanitary environment (*n* 7), the active living environment (*n* 26) and the food environment (*n* 12). This survey was completed by 27 162 Canadians living in the country’s seventeen most densely populated CMAs. Data were collected between October and December 2020. Sample size objectives were established at 1200 participants for most CMAs. The THEPA survey dataset is nationally representative of the urban Canadian population, as data were weighted as a function of sex, age and education, based on data from the 2016 Canadian census^(26)^. Post-stratification weights were also applied at the CMA level to account for CMA size differences.

### Eligibility criteria and recruitment

As the survey was available in either English or French, eligibility criteria included speaking/reading English and/or French. Participants had to be at least 18 years of age and minimally provide residential forward sortation area information. To complete the survey, participants were contacted either by email (based on previous enrolment in survey firm panels) or by phone (based on random digit dialling).

### Measurements

#### Acceptability

Acceptability was measured by asking participants to rate their level of agreement with the implementation of each intervention within their area of residence, that is, the area within a 15-min walking distance from one’s dwelling. The three policies directly pertaining to the RFE were stated in the THEPA survey as follows:

1. Change the usual side dish in restaurants for a healthier option like salad instead of fries.
2. Impose municipal regulations to limit fast-food outlets around schools.
3. Eliminate the offer of chips, candy and other unhealthy foods in restaurants, cafeterias and vending machines in municipal buildings like arenas and recreation centres.

For each policy, a 4-point rating scale was provided. Response options were ‘completely disagree’, ‘somewhat disagree’, ‘somewhat agree’ and ‘completely agree’. Participants were also given the possibility to select the ‘I don’t know/ I prefer not to respond’ case. Acceptability responses were then dichotomised into ‘complete agreement’ and ‘other’. The ‘complete agreement’ category encompassed ‘completely agree’ responses, whereas the ‘other’ category was comprised of ‘somewhat agree’, ‘completely disagree’ and ‘somewhat disagree’ responses. Dichotomising the outcome variable as such was underpinned by Kingdon’s theory highlighting the importance of high acceptability levels, compared with lower acceptability levels, to precipitate RFE policy change. As for the ‘I don’t know/I prefer not to respond’ cases, these were considered missing.

#### Sociodemographic characteristics

Sociodemographic characteristics pertained to gender (‘man’ or ‘woman’), age (‘18–34 years old’, ‘35–54 years old’ or ‘55 years old and over’), highest educational attainment (‘high school and less’, ‘trade school or junior college’ or ‘university’), gross household annual income (‘less than $40 000’, ‘$40 000–$79 999’, ‘$80 000–$119 999’ or ‘$120 000 and more’), immigrant status (‘born in Canada’, ‘born in a high-income country (HIC) other than Canada’ or ‘born in a low- or middle-income country (LMIC)’) and Indigenous status (‘Indigenous’ or ‘non-Indigenous’). For an in-depth examination of how sociodemographic questions were formulated in the THEPA survey and for a better understanding of how the responses to the latter questions were recoded, see Lambert-De Francesch *et al.* (2024)^(17)^.

#### Health characteristics

Four health-related variables from the THEPA survey were deemed relevant for this study: self-reported weight, self-reported height, frequency of consuming restaurant-prepared foods and perceived health status. To estimate weight, participants were asked ‘How much do you weigh?’. Participants could respond either in pounds or kilograms. To assess height, participants were asked ‘How tall are you?’. Participants could respond in either feet/inches or in metres. For both weight and height questions, an ‘I don’t know/I prefer not to answer’ option was available. Using the provided anthropometric data, BMI scores were computed and recoded in the following weight status cutoffs: ‘underweight’ (< 18·50 kg/m^2^), ‘normal weight’ (18·50–24·99 kg/m^2^), ‘overweight’ (25·00–29·99 kg/m^2^) and ‘obese’ (≥ 30·00 kg/m^2^)^(27)^. As a marker of dietary practices, participants were asked ‘During an average week, how often do you eat in restaurants?’, with response options being ‘every day’, ‘a few times a week’, ‘once a week’, ‘less than once a week’, ‘never’ and ‘I don’t know/I prefer not to answer’. To assess health status, participants were asked ‘Compared to other people of your age, would you say your health is ‘excellent’, ‘very good’, ‘good’, ‘fair’, ‘poor’ or ‘I don’t know/I prefer not to answer’. For both restaurant food consumption frequency and perceived health status variables, no response options were recoded, as this enabled a more fine-grained analysis of distinct categories. For all health characteristic variables, ‘I don’t know/I prefer not to answer’ responses were considered missing.

### Data cleaning and imputing

To obtain BMI scores that would better mirror those extracted from measured data, modifications were made to the self-reported anthropometric data. The first step consisted of exclusively retaining height and weight values that were deemed biologically plausible. Height values that were considered biologically plausible ranged from 1·12 m (3′ 8″) to 2·29 m (7′ 6″), whereas weight values that were considered biologically plausible ranged from 34·09 kg (75 lbs) to 454·50 kg (1000 lbs). All values outside of these ranges were considered missing. The second data quality check consisted of creating z-scores for both height and weight to remove any potential outliers. Cases with standardised z-scores greater than 3·29 were considered potential outliers and were thus considered missing^(28)^. The remaining values were used to compute BMI scores. These BMI scores underwent a third quality examination procedure. BMI values less than 12·00 were considered biologically implausible. The fourth and final step to ensure that BMI values best reflected measured height and weight entailed applying gender-specific correction equations to BMI scores to curb social desirability biases^(29)^. Corrected BMI scores were then recoded into the above-mentioned BMI categories, that is, ‘underweight’, ‘normal weight’, ‘overweight’ or ‘obese’.

To address potential issues that may arise from using incomplete survey datasets, imputed datasets (*n* 10) were created using multiple imputation^(28)^. Predictive mean matching methods were employed to identify case ‘donors’ that had similar predicted values to those of the missing value^(30)^. For each imputed dataset, one value among the donor case pool (*n* 5 potential donors) was randomly assigned to the missing data case. The ten datasets were pooled into one large dataset and used for statistical analyses.

### Data analyses

Descriptive analyses were conducted to highlight participants’ sociodemographic and health profiles. Among the non-weighted/non-imputed, weighted/non-imputed and weighted/imputed datasets, descriptive analyses for sociodemographic characteristics were only conducted on the weighted/imputed dataset to avoid duplication. Sociodemographic information based on non-weighted/non-imputed and weighted/non-imputed data has been reported and published elsewhere^(17)^. It was deemed important to present sociodemographic characteristics from the weighted/imputed dataset since these data slightly differ from what is presented in the aforementioned publication. As for the health variables of interest, given that these data have not been previously published, descriptive analyses were conducted on all three datasets. Up to this point, all steps were carried out using SPSS version 28.

Logistic multilevel modelling analyses were then performed using HLM 8·0 software. The latter statistical technique was deemed relevant for the current study objectives, as analysing the data via logistic multilevel modelling respects the data’s nested structured (i.e. individuals nested within higher-level units, CMAs). Failure to recognise the hierarchical structure of the data may result in an underestimation of standard error values, incorrectly leading to narrower confidence intervals and greater risk of committing a type 1 error^(31)^.

A total of three sets of analyses were performed using the weighted/imputed data. First, bivariate logistic multilevel regressions were performed to assess the distinct associations between individual-level variables (i.e. sociodemographic characteristics and health characteristics) and the acceptability levels of three RFE policies. Second, multivariate logistic multilevel regressions were conducted to investigate the associations between the three health characteristics, evaluated jointly, and the acceptability levels of each policy (model 1). Third, the latter examinations were replicated using the same type of analysis, this time adjusting for sociodemographic variables (model 2). Across all analyses, health and sociodemographic variables were modelled as level-1 fixed effects, meaning that their influence was assumed to be constant across CMAs. CMA of residence was considered a nesting factor (i.e. level-2 factor), reflecting the hierarchical nature of the dataset. Finally, odds ratios (ORs) and 95 % confident intervals (CIs) are reported for all analyses.

## Results

### Participant characteristics

Table 1 presents the sociodemographic and health characteristics of participants. According to the weighted/imputed dataset analyses, the sociodemographic characteristics with the highest prevalence included identifying as women (51·8 %), being aged 55 years and older (35·4 %), having a high school education level or lower (41·2 %), having a gross household income of $40 000–$79 999 per year (31·8 %), being born in Canada (77·8 %) and not self-identifying as Indigenous (94·5 %). The most prevalent weight status was the overweight category, accounting for 33·9 % of responses in the weighted/imputed dataset. The most frequent perceived health status was the ‘good’ category, representing 36·2 % of responses in the weighted/imputed dataset. For the frequency of consuming restaurant-prepared foods variable, the less than once a week category was most observed (43·2 % of responses).

**Table 1.** Sociodemographic and health characteristics of participants partaking in the *Targeting Healthy Eating and Physical Activity* survey (*n* 27 162). Data were collected between October and December 2020.

Individual characteristics	Unweighted/ non-imputed dataset		Weighted/non-imputed dataset	Weighted/imputed dataset
	*n*	%	%	%
Gender				
Male	–		–	48·2
Female	–		–	51·8
Other	–		–	–
Missing	–		–	–
Age				
18–34 years	–		–	29·6
35–54 years	–		–	35·0
55 years or older	–		–	35·4
Missing	–		–	–
Education				
High school or less	–		–	41·2
Trade school or junior college	–		–	29·6
University	–		–	29·1
Missing	–		–	–
Annual family income before taxes				
Less than $40 000	–		–	25·6
Between $40 000 and $79 999	–		–	31·8
Between $80 00 and $119 999	–		–	23·6
$120 000 and over	–		–	19
Missing	–		–	–
Country of birth				
Canada	–		–	77·8
High-income country outside of Canada	–		–	7·2
Low- or middle-income country	–		–	14·9
Missing	–		–	–
Self-reported Indigenous status				
Non-Indigenous	–		–	94·5
Indigenous	–		–	5·5
Missing	–		–	–
BMI				
Underweight	464	1·7	2·1	4·9
Normal weight	7471	27·5	28·0	31·0
Overweight	8045	29·6	28·5	33·9
Obese	6654	24·5	23·4	30·2
Missing	4528	16·7	18·0	–
Perceived health status				
Excellent	3603	13·3	13·2	13·3
Very good	8950	33·0	31·0	31·2
Good	9633	35·5	36·0	36·2
Fair	3735	13·8	14·9	15·2
Poor	1029	3·8	4·1	4·2
Missing	212	0·8	0·9	–
Average frequency of consuming foods in restaurants				
Every day	403	1·5	2·1	2·1
A few times a week	3312	12·2	13·1	13·1
Once a week	5664	20·9	20·8	20·9
Less than once a week	12 075	44·5	43·2	43·2
Never	5239	19·3	20·6	20·7
Missing	469	1·7	0·2	–

### Variables associated with acceptability of targeted policies

Associations between RFE policy acceptability, sociodemographic variables and health variables are presented in Table 2. Unless specified otherwise, ORs and 95 % CIs described in the text below are reflective of those extracted from the final model, model 2.

**Table 2.** Odds ratios and 95 % confidence intervals for being in complete agreement with each restaurant food environment policy according to the sociodemographic and health characteristics of participants partaking in the *Targeting Healthy Eating and Physical Activity* survey (*n* 27 162). Data were collected between October and December 2020. Bolded results with a † superscript symbol are statistically significant.

	Change the usual side dish						Limit fast-food outlets around schools						Eliminate the offer of unhealthy foods					
Model OR (95 % CI)	Bivariate associations		Model 1: all health indicators		Model 2: model 1 adjusted for sociodemographic variables		Bivariate associations		Model 1: all health indicators		Model 2: model 1 adjusted for sociodemographic variables		Bivariate associations		Model 1: all health indicators		Model 2: model 1 adjusted for sociodemographic variables	
	OR	95 % CI	OR	95 % CI	OR	95 % CI	OR	95 % CI	OR	95 % CI	OR	95 % CI	OR	95 % CI	OR	95 % CI	OR	95 % CI
Gender																		
Man	1·00				1·00		1·00				1·00		1·00				1·00	
Woman	**1·51** †	1·43, 1·60			**1·57** †	1·49, 1·67	**1·18** †	1·11, 1·25			**1·19** †	1·13, 1·27	**1·17** †	1·10, 1·25			**1·21** †	1·13, 1·29
Age																		
18–34 years	0·91	0·82, 1·01			**0·87** †	0·80, 0·94	**0·85** †	0·80, 0·92			**0·85** †	0·79, 0·92	**0·87** †	0·80, 0·94			**0·82** †	0·75, 0·90
5–54 years	0·99	0·92, 1·06			1·01	0·94, 1·09	0·96	0·89, 1·03			0·99	0·91, 1·06	**0·91** †	0·85, 0·99			**0·91** †	0·84, 0·99
55 years or older	1·00				1·00		1·00				1·00		1·00				1·00	
Education																		
High school or less	0·94	0·88, 1·00			0·93	0·86, 1·00	**0·84** †	0·78, 0·90			**0·80** †	0·74, 0·86	**0·79** †	0·73, 0·85			**0·78** †	0·72, 0·85
Trade school or junior college	1·03	0·96, 1·11			1·03	0·96, 1·11	**0·90** †	0·83, 0·97			**0·89** †	0·82, 0·96	**0·92** †	0·85, 0·99			0·93	0·86, 1·01
University	1·00				1·00		1·00				1·00		1·00				1·00	
Annual family income before taxes																		
Less than $40 000	**1·14** †	1·05, 1·25			**1·15** †	1·05, 1·27	**1·11** †	1·03, 1·20			**1·13** †	1·04, 1·22	**1·15** †	1·06, 1·25			**1·19** †	1·09, 1·30
Between $40 000 and $79 999	1·00				1·00		1·00				1·00		1·00				1·00	
Between $80 000 and $119 999	**0·91** †	0·84, 0·99			**0·91** †	0·84, 0·99	**0·90** †	0·83, 0·98			**0·90** †	0·82, 0·97	0·92	0·84, 1·01			0·91	0·83, 1·01
$120 000 and over	0·95	0·87, 1·05			0·92	0·84, 1·01	0·98	0·90, 1·07			0·91	0·83, 1·01	1·05	0·95, 1·16			0·98	0·89, 1·08
Country of Birth																		
Canada	1·00				1·00		1·00				1·00		1·00				1·00	
High-income country other than Canada	**1·37** †	1·21, 1·55			**1·32** †	1·16, 1·50	**1·22** †	1·09, 1·36			**1·14** †	1·02, 1·29	**1·44** †	1·24, 1·66			**1·34** †	1·15, 1·57
Low- or middle-income country	**1·28** †	1·18, 1·39			**1·26** †	1·15, 1·37	**1·09** †	1·01, 1·18			1·05	0·97, 1·15	**1·37** †	1·26, 1·50			**1·32** †	1·20, 1·45
Self-reported indigenous status																		
No	1·00				1·00		1·00				1·00		1·00				1·00	
Yes	**1·61** †	1·43, 1·81			**1·55** †	1·36, 1·76	**1·46** †	1·29, 1·65			**1·45** †	1·27, 1·65	**1·57** †	1·39, 1·78			**1·51** †	1·33, 1·72
BMI																		
Underweight	0·91	0·75, 1·10	0·93	0·76, 1·13	0·93	0·77, 1·14	0·88	0·73, 1·07	0·90	0·74, 1·01	0·91	0·75, 1·11	0·97	0·77, 1·22	0·99	0·79, 1·25	1·00	0·80, 1·26
Normal weight	1·00		1·00		1·00		1·00		1·00		1·00		1·00		1·00		1·00	
Overweight	0·92	0·85, 1·00	0·95	0·88, 1·03	1·00	0·92, 1·09	0·98	0·91, 1·06	1·01	0·94, 1·09	1·02	0·95, 1·11	0·95	0·87, 1·03	0·98	0·90, 1·07	0·99	0·91, 1·09
Obese	**0·90** †	0·81, 0·99	0·99	0·90, 1·09	1·04	0·93, 1·15	**0·91** †	0·84, 0·99	1·00	0·91, 1·09	1·01	0·92, 1·11	**0·88** †	0·81, 0·96	0·99	0·91, 1·07	1·01	0·92, 1·11
Perceived health status																		
Excellent	**2·11** †	1·94, 2·30	**2·05** †	1·88, 2·24	**2·12** †	1·94, 2·32	**1·99** †	1·82, 2·17	**1·92** †	1·75, 2·10	**1·93** †	1·76, 2·11	**2·26** †	2·06, 2·47	**2·15** †	1·96, 2·36	**2·15** †	1·96, 2·36
Very good	**1·32** †	1·23, 1·42	**1·32** †	1·23, 1·42	**1·36** †	1·26, 1·45	**1·32** †	1·23, 1·42	**1·32** †	1·23, 1·42	**1·32** †	1·23, 1·42	**1·37** †	1·27, 1·49	**1·38** †	1·27, 1·49	**1·37** †	1·27, 1·49
Good	1·00		1·00		1·00		1·00		1·00		1·00		1·00		1·00		1·00	
Fair	1·02	0·93, 1·13	1·02	0·92, 1·12	0·98	0·89, 1·09	1·08	0·99, 1·18	1·07	0·98, 1·18	1·05	0·96, 1·15	1·09	0·99, 1·20	1·08	0·98, 1·19	1·07	0·96, 1·18
Poor	**1·17** †	1·01, 1·36	1·13	0·98, 1·32	1·08	0·93, 1·26	**1·30** †	1·12, 1·52	**1·24** †	1·07, 1·45	**1·20** †	1·03, 1·40	1·08	0·90, 1·28	1·03	0·86, 1·23	1·02	0·85, 1·21
Average frequency of consuming foods in restaurants																		
Every day	**2·01** †	1·59, 2·54	**1·68** †	1·32, 2·14	**1·69** †	1·33, 2·16	**2·24** †	1·85, 2·71	**1·93** †	1·59, 2·34	**1·92** †	1·58, 2·34	**2·70** †	2·20, 3·32	**2·24** †	1·82, 2·77	**2·22** †	1·79, 2·75
A few times a week	1·07	0·97, 1·18	1·05	0·95, 1·16	1·06	0·96, 1·17	1·01	0·91, 1·12	1·00	0·90, 1·10	0·99	0·90, 1·10	1·01	0·91, 1·13	1·00	0·89, 1·11	0·99	0·89, 1·11
Once a week	1·00		1·00		1·00		1·00		1·00		1·00		1·00		1·00		1·00	
Less than once a week	0·99	0·91, 1·07	1·00	0·92, 1·08	0·94	0·87, 1·02	1·05	0·97, 1·13	1·05	0·97, 1·14	1·02	0·94, 1·11	1·02	0·93, 1·11	1·03	0·94, 1·12	1·00	0·91, 1·09
Never	**1·23** †	1·12, 1·34	**1·23** †	1·13, 1·35	**1·14** †	1·04, 1·25	**1·46** †	1·34, 1·59	**1·46** †	1·34, 1·60	**1·38** †	1·26, 1·52	**1·36** †	1·24, 1·50	**1·37** †	1·25, 1·51	**1·30** †	1·18, 1·43

† Statistically significant.

#### Changing side dish defaults

For the change the usual side dish policy, in terms of sociodemographic variables, across all analyses, greater odds of being in complete agreement with the selected policy were observed among women (OR: 1·57, 95 % CI: 1·49, 1·67), those with household earnings that were less than $40 000 per annum (OR: 1·15, 95 % CI: 1·05, 1·27), those born in a HIC other than Canada (OR: 1·32, 95 % CI: 1·16, 1·50), those born in a LMIC (OR: 1·26, 95 % CI: 1·15, 1·37) and those with an Indigenous status (OR: 1·55, 95 % CI: 1·36, 1·76), in contrast to men, those with household earnings of $40 000–$79 999 per annum, non-immigrants and non-Indigenous participants. A lower likelihood of being in complete agreement with the selected policy was observed among those aged 18–34 years old (in model 2 only, OR: 0·87, 95 % CI: 0·80, 0·94) and those with household earnings of $80 000–$119 999 per annum (in all analyses, OR: 0·91, 95 % CI: 0·84, 0·99), compared with those aged 55 years and over and those with annual household incomes of $40 000–$79 999.

As for health characteristics, only in the bivariate analysis was one BMI category associated with complete agreement levels. Indeed, compared with those with a normal weight, those falling within the obese category had a lower likelihood of being in complete agreement with the policy (OR: 0·90, 95 % CI: 0·81, 0·99). Furthermore, in contrast to those with a good perceived health status, across all analyses, both excellent (OR: 2·12, 95 % CI: 1·94, 2·32) and very good (OR: 1·32, 95 % CI: 1·23, 1·42) perceived health statuses were associated with a greater likelihood of expressing complete agreement for the selected policy. A poor health status was also associated with greater odds of being in complete agreement with the policy than a good health status, but this was unique to the bivariate association (OR: 1·17, 95 % CI: 1·01, 1·36). In all analyses, greater odds of being in complete agreement with the targeted policy were also observed among those consuming restaurant-prepared foods every day and those never consuming restaurant-prepared foods, contrary to those eating restaurant-prepared foods once a week, with respective ORs being 1·69 (95 % CI: 1·33, 2·16) and 1·14 (95 % CI: 1·04, 1·25).

#### Limiting fast foods around schools

Analogous to the first policy, independently of the type of analysis, women, those with household incomes of less than $40 000, those born in a HIC other than Canada, those with an Indigenous status, those with an excellent and very good perceived health status and those consuming restaurant-prepared food every day or never were all more likely to be in complete agreement with the targeted policy than men, those in the $40 000–$79 999/year income category, those born in Canada, those with a non-Indigenous status, those with a good perceived health status and those consuming restaurant-prepared foods once per week. Respectively, ORs were 1·19 (95 % CI: 1·13, 1·27) for women, 1·13 (95 % CI: 1·04, 1·22) for those earning less than $40 000 per annum, 1·14 (95 % CI: 1·02, 1·29) for those born in a HIC other than Canada, 1·45 (95 % CI: 1·27, 1·65) for those with an Indigenous status, 1·93 (95 % CI: 1·76, 2·11) for those with an excellent perceived health status, 1·32 (95 % CI: 1·23, 1·42) for those with a very good perceived health status, 1·92 (95 % CI: 1·58, 2·34) for those that eat restaurant-prepared foods every day and 1·38 (95 % CI: 1·26, 1·52) for those that never eat restaurant-prepared foods. Once more, across analyses, those earning between $80 000 and $119 999 had lower odds of being in complete agreement with the targeted policy than those within the $40 000–$79 999 income bracket (OR: 0·90, 95 % CI: 0·82, 0·97). Akin to the first policy, there was no statistically significant association between BMI and acceptability levels, except for those in the obese category in the bivariate analysis, OR: 0·91 (95 % CI: 0·84, 0·99).

Despite the aforementioned similarities with the previous policy, selected differences are observed. Contrary to the first policy, those aged between 18 and 34 years old had lower odds of being in complete agreement with the targeted policy in all analyses than those aged 55 years and older (OR: 0·85, 95 % CI: 0·79, 0·92). Also, unlike the first policy, across analyses, those with a high school level training (or less) and those with a trade/junior college training had a lower likelihood of completely agreeing with the selected policy than those with a university training, with ORs being 0·80 (95 % CI: 0·74, 0·86) and 0·89 (95 % CI: 0·82, 0·96), respectively. Furthermore, unique to this policy, being born in a LMIC was only associated with greater odds of being in complete agreement with the selected policy in the bivariate association (OR: 1·09, 95 % CI: 1·01, 1·18), and this is comparative to those born in Canada. Finally, differing from the first policy, associations between poor perceived health status and complete agreement levels remained statistically significant, irrespective of the analysis, OR: 1·20, 95 % CI: 1·03, 1·40.

#### Eliminating unhealthy food

In line with the two previous policies, women (OR: 1·21, 95 % CI: 1·13, 1·29), those with household earnings of less than $40 000 per year (OR: 1·19, 95 % CI: 1·09, 1·30), immigrants from a HIC other than Canada (OR: 1·34, 95 % CI: 1·15, 1·57), those with an Indigenous status (OR: 1·51, 95 % CI: 1·33, 1·72), those with an excellent health status (OR: 2·15, 95 % CI: 1·96, 2·36), those with a very good health status (OR: 1·37, 95 % CI: 1·27, 1·49), those consuming restaurant-prepared foods every day (OR: 2·22, 95 % CI: 1·79, 2·75) and those never consuming restaurant-prepared foods (OR: 1·30, 95 % CI: 1·18, 1·43) all had greater odds of expressing complete agreement with the targeted policy than those in the reference category. Unique to this policy, in all analyses, both those aged 18–34 years old and those aged 35–54 years old had lower odds of reporting complete agreement levels compared with those aged 55 years and over, with respective ORs being 0·82 (95 % CI: 0·75, 0·90) and 0·91 (95 % CI: 0·84, 0·99). Similarly to the second policy (i.e. limit fast-food outlets around schools), across analyses, having a high school level (or less) training was associated with lower odds of being in complete agreement with the policy (OR: 0·78, 95 % CI: 0·72, 0·85). As for those with a trade school/junior college education, a significant association was only observed in bivariate associations, where participants with the latter level of education had lower odds of being in complete agreement with the targeted policy than those with a university degree (OR: 0·92, 95 % CI: 0·85, 0·99). In all analyses, those from a LMIC displayed greater odds of being in complete agreement with the policy than their Canadian-born counterparts (OR: 1·32, 95 % CI: 1·20, 1·45). Once more, BMI was mostly not associated with acceptability levels. Only findings from the bivariate analysis showed that those with BMIs falling in the obese category had lower odds of expressing complete agreement levels with the targeted policy than those with a normal weight (OR: 0·88, 95 % CI: 0·81, 0·96).

**Figure 1.**
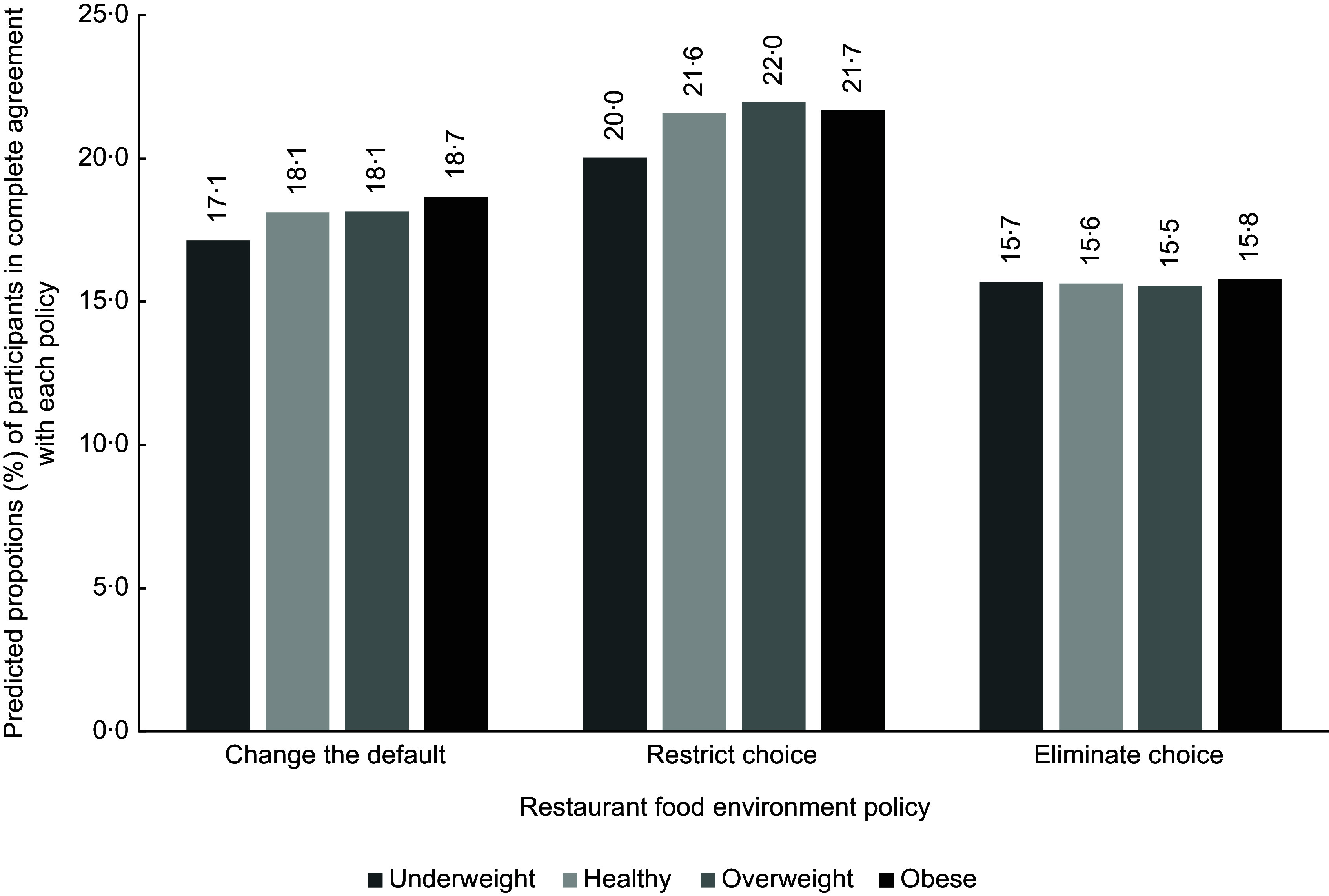
Predicted proportions, based on model 2, of participants being in complete agreement with each restaurant food environment policy according to BMI category. Predicted proportions are illustrated for individuals corresponding to the following reference categories (i.e. men, aged 55 years and over, with university training, with an annual household income of $40 000–$79 999, that were born in Canada, that do not have an Indigenous status, that have a good perceived health status and that eat restaurant-prepared foods once per week).

**Figure 2.**
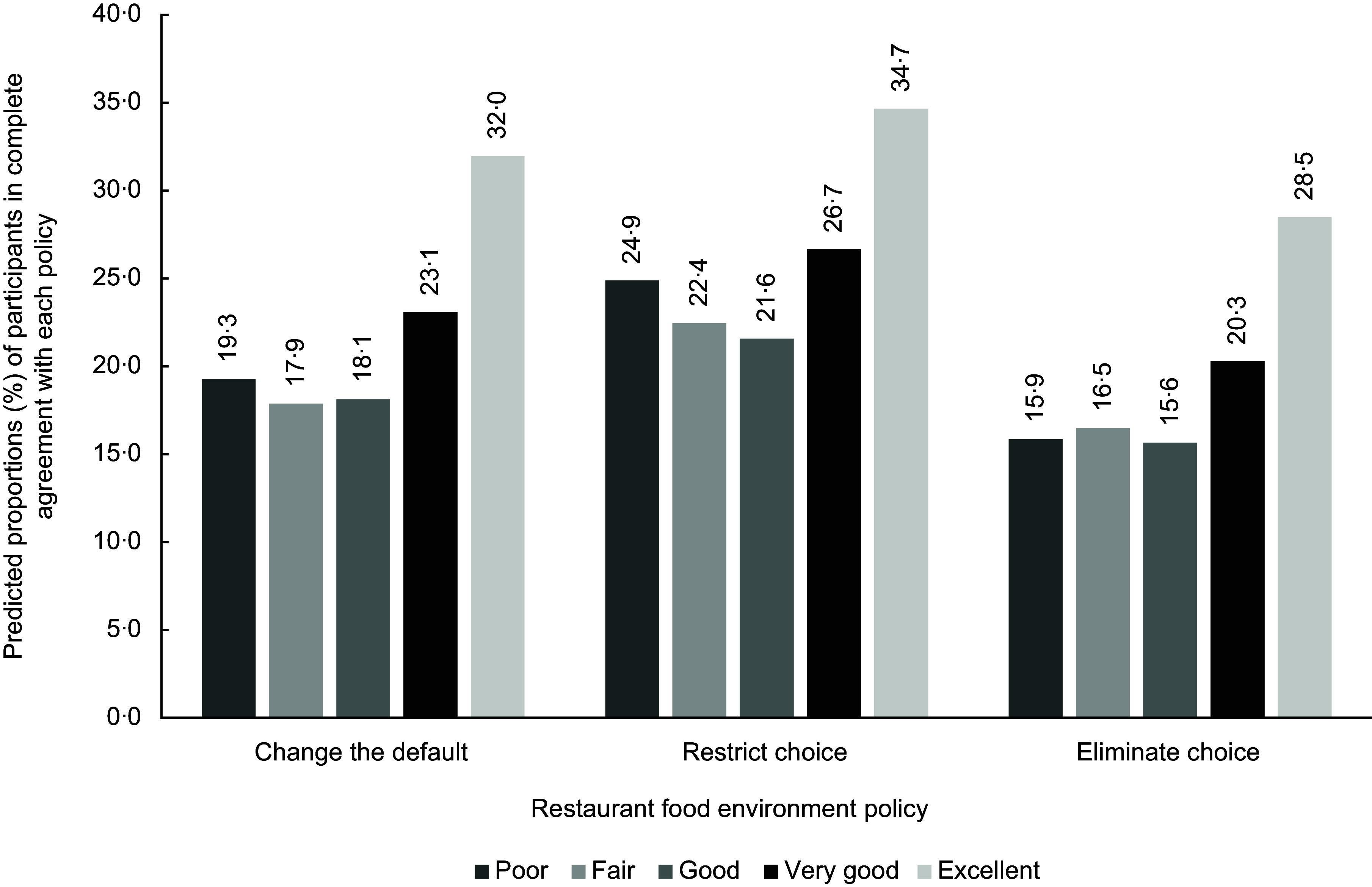
Predicted proportions, based on model 2, of participants in complete agreement with each restaurant food environment policy according to perceived health status. Predicted proportions are illustrated for individuals corresponding to following reference categories (i.e. men, aged 55 years and over, with university training, with an annual household income of $40 000–$79 999, that were born in Canada, that do not have an Indigenous status, that have a normal weight and that eat restaurant-prepared foods once per week).

**Figure 3.**
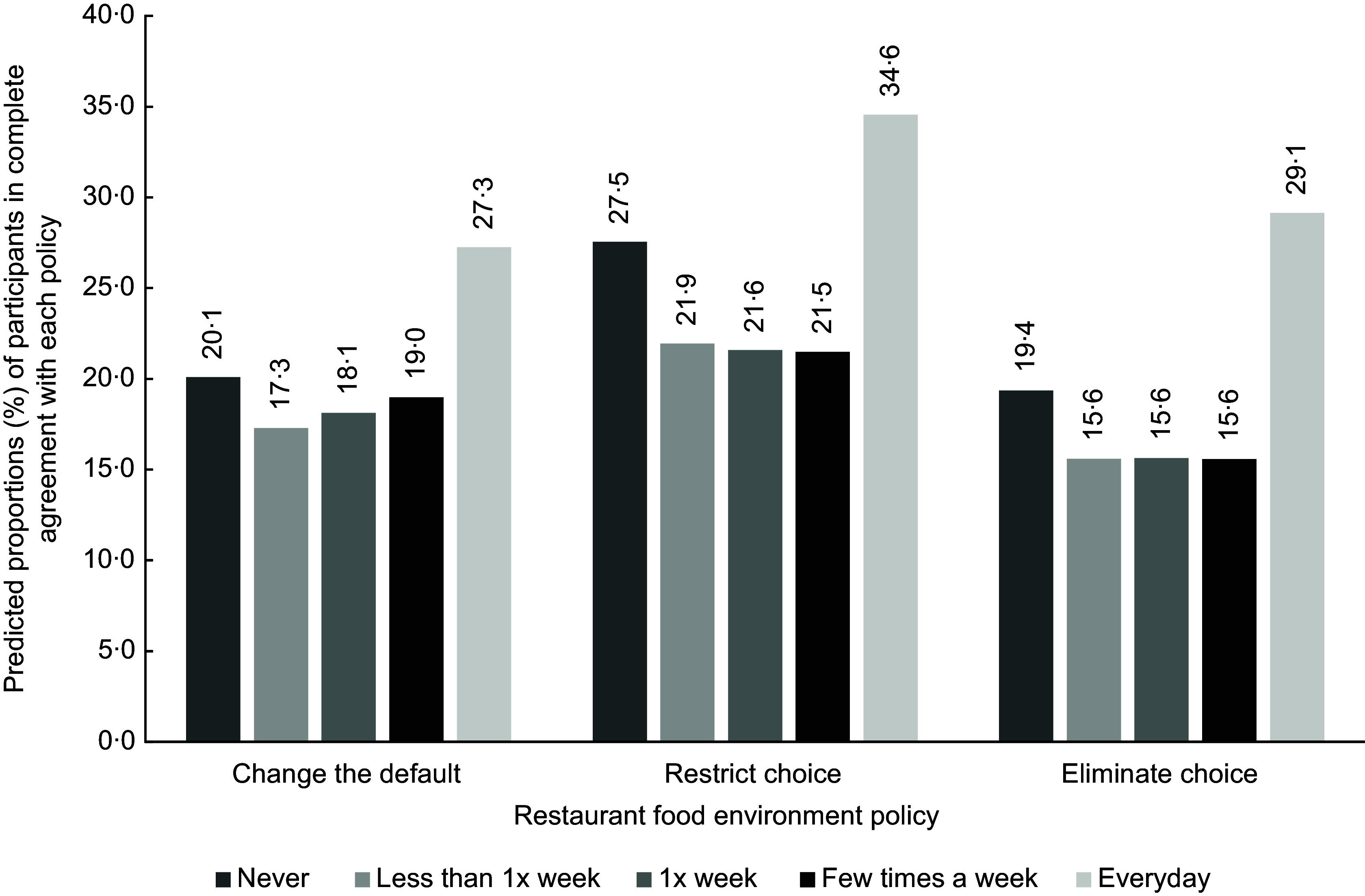
Predicted proportions, based on model 2, of participants in complete agreement with each restaurant food environment policy according to frequency of consuming restaurant-prepared foods. Predicted proportions are illustrated for individuals corresponding to the following reference categories (i.e. men, aged 55 years and over, with university training, with an annual household income of $40 000–$79 999, that were born in Canada, that do not have an Indigenous status, that have a normal BMI and that have a good perceived health status).

To better illustrate the pattern of findings for all three policies, histograms were created. Figures 1, 2 and 3 portray predicted proportions of those in complete agreement with each RFE policy according to health characteristics. Predicted proportions were based on model 2 and were calculated for those whose sociodemographic characteristics matched the reference category. The predicted proportions were also calculated based on reference categories of the unexamined health variables. The 95 % CIs are not presented in the figures since estimates are based on predicted values derived from equations, contrary to estimates that are based on raw data.

As shown in Fig. 1, little variation in terms of predicted proportions exists across BMI categories, and this for the three policies. In Fig. 2, across policies, those with an excellent and very good perceived health status discernibly had greater predicted proportions of being in complete agreement with the policies than those with a good health status. For the restrict choice policy, greater predicted proportions were also apparent for those with a poor health status, compared with the reference category. Finally, for Fig. 3, those on opposite ends of the restaurant frequency consumption spectrum (i.e. those in the never and every day category) visually had greater predicted proportions of being in complete agreement than those consuming restaurant-prepared foods once a week.

## Discussion

The purpose of this study was to examine associations between selected individual health characteristics and complete agreement levels of three distinct RFE policies among urban Canadian adults. Findings relating to the sociodemographic correlates of acceptability revealed that, based on the final model, women, those with household incomes inferior to $40 000 per annum, those who were born in a HIC other than Canada and those with an Indigenous status were more likely to be in complete agreement with all policies, compared with men, those with $40 000–$79 999 household annual incomes, those born in Canada and those who are not Indigenous. As for those aged between 18 and 34 years old, these individuals were less likely to be in complete agreement with all policies, compared with those aged 55 years old and over. As for health-related findings, results indicated that BMI was generally not associated with acceptability of RFE policies. Across all three policies, those with either an excellent or a very good health status were more likely to express complete agreement with RFE policies than those with a good health status. For selected policies, those with a poor health status were also more likely to be in complete agreement with the examined policies than those with a good health status. For all three policies, those consuming restaurant-prepared foods every day and never had greater odds of being in complete agreement with RFE policies than those who reported consuming these foods once per week.

As hypothesised elsewhere^(17)^, greater acceptability levels among women may be related to their greater health consciousness level^(32)^. As for greater acceptability levels among those with the lowest income category, this observation may be related to their financial limitations when it comes to purchasing nutritious foods^(33,34)^. Higher levels of acceptability among those born in a HIC other than Canada may be related to a higher level of familiarity with healthier RFE in their birth country^(17)^. As for results regarding Indigenous status, few acceptability studies have included this variable in their research, and further study is required to gain insight on these links^(22)^. Finally, lower acceptability levels observed among younger participants (18–34 years old) may be related to a lower level of awareness of the toll diet-related diseases may have as one ages^(15)^. In all cases, the above-described rationale behind associations is only speculative and requires further investigation^(17)^.

When comparing results to studies that have examined associations between health characteristics and acceptability levels of RFE policies, our results regarding the lack of a statistically significant association between BMI (in multivariate analyses) and acceptability are similar to those of Bhawra *et al.* (2018)^(22)^. Similar to the current findings, these authors report no statistically significant association between BMI and support for selected RFE policies, such as fast-food zoning policies around schools among Canadians aged 16–30 years old. Results are also somewhat similar to those of Robles and Kuo (2017) who observed no statistically significant association between perceived weight status and acceptability for limiting/restricting food policies, such as limiting the count of fast foods within each community^(21)^. Yet, the commonalities between our findings and those of the latter authors may be limited by the fact that the latter authors created a five-item composite score for its limiting/restrictive policies, where only one item specifically related to our scope of interest (i.e. fast-food zoning policies).

To the best of our knowledge, only one other study has examined the links between perceived health status and RFE policy acceptability^(21)^. These authors found no statistically significant association between their limiting/restrictive food policies and perceived health status, findings that are contrary to ours. More research is needed to reconcile these discrepant findings.

Regarding restaurant frequency consumption variables, Robles and Kuo’s (2017) study remains the only point of comparison^(21)^. These authors found no statistically significant association between frequency of consuming restaurant-prepared foods and acceptability of limiting/restricting food policies, like fast-food zoning restrictions. Although our results regarding restaurant food consumption frequency are not analogous to those of the latter study, selected results do converge with broader observations^(15)^. According to prior work linking personal impact of policies and acceptability levels of these policies^(15,18,19)^, those who never consume restaurant-prepared foods would be more likely to be in complete agreement with RFE policies than those consuming these foods once per week, a tendency that was confirmed by this study. Contrary to this general tendency are, however, our findings regarding those who consume these foods daily. Greater acceptability levels observed among the latter population segment may signal a desire for healthier restaurant food choices. More research is however needed on the matter.

In terms of implications, the findings from this study may help advance knowledge regarding the agenda setting process required for RFE policy enactment. Identifying the individual health characteristics associated with RFE policy acceptability creates an opportunity for further discussion on the rationale behind these health-related differences in acceptability. Understanding the underlying drivers of acceptability levels according to health characteristics may help orient communication campaigns aimed at informing the public on the benefits of RFE policies and invalidating unfounded beliefs that may be hindering RFE policy acceptability levels across population subgroups. In line with the *Multiple Streams Framework*, a more favourable political climate, shaped by high acceptability levels, may ultimately help propel policy entrepreneurs’ efforts in advocating for evidence-based RFE policies, thus contributing to overweight/obesity mitigation efforts.

Methodological strong points pertain to the numerous steps taken to ensure the development of biologically plausible and likely valid BMI scores as well as the steps taken to conduct multiple imputations and logistic multilevel modelling analyses. Despite these strengths, there are certain caveats in this study’s design. As mentioned previously, self-reporting of height and weight is subject to social desirability biases, which may lead to an overestimation of height and an underestimation of weight parameters^(29)^. Furthermore, BMI scores fail to account for body composition and body fat distribution^(35)^. Finally, an important limitation of this study is the fact that acceptability ‘I don’t know/I prefer not to respond’ case options were recoded as missing. Further study could focus on the characteristics of participants who selected this response alternative.

### Conclusion

This study contributed to bridging current gaps in the food policy acceptability literature as key health characteristics associated with complete agreement levels for three RFE policies have been identified. The findings from this study lay the groundwork for further studies aiming to explore the underlying motives of health-related acceptability levels. Depicting a clearer portrait of RFE policy acceptability levels in urban Canada may help advance the RFE policy scene, necessary for addressing pressing public health nutrition issues.
